# Speech Processing Disorder in Neural Hearing Loss

**DOI:** 10.1155/2012/206716

**Published:** 2012-12-06

**Authors:** Joseph P. Pillion

**Affiliations:** ^1^Department of Audiology, Kennedy Krieger Institute, 801 North Broadway, Baltimore, MD 21205, USA; ^2^Department of Physical Medicine and Rehabilitation, Johns Hopkins University School of Medicine, Baltimore, MD 21205, USA

## Abstract

Deficits in central auditory processing may occur in a variety of clinical conditions including traumatic brain injury, neurodegenerative disease, auditory neuropathy/dyssynchrony syndrome, neurological disorders associated with aging, and aphasia. Deficits in central auditory processing of a more subtle nature have also been studied extensively in neurodevelopmental disorders in children with learning disabilities, ADD, and developmental language disorders. Illustrative cases are reviewed demonstrating the use of an audiological test battery in patients with auditory neuropathy/dyssynchrony syndrome, bilateral lesions to the inferior colliculi, and bilateral lesions to the temporal lobes. Electrophysiological tests of auditory function were utilized to define the locus of dysfunction at neural levels ranging from the auditory nerve, midbrain, and cortical levels.

## 1. Introduction

Audiological evaluations typically involve assessment of sensitivity for pure tones which is summarized on an audiological record or audiogram. For most patients, the demonstration of normal peripheral auditory sensitivity suggests that auditory function is most likely adequate for speech and language development and communication; a normal audiogram is typically taken as indicative of normal auditory function for educational and communicative purposes. However, for many patients with neurological disease or dysfunction, a normal audiogram does not predict how the patient functions in every day listening conditions [1]. In the present paper, cases will be presented in which the audiogram did not predict the marked deficits in speech processing experienced by the patients. The neural mechanisms underlying the patient's, hearing and speech processing deficits are reviewed in detail.

**Case 1**

**Figure 1:**
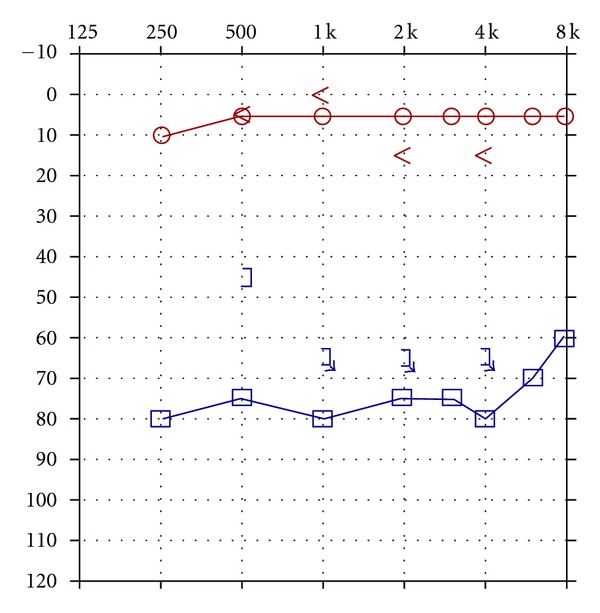
Audiogram depicting normal auditory sensitivity for the right ear and a hearing loss of severe degree and sensorineural origin for the left ear.

Patient CF was a 7 year old who was thought to have had normal hearing until he failed a school screening and a subsequent screening in his pediatrician's office for his left ear. He was functioning above grade level academically. Speech articulation was good and there were no behavioral concerns. He was the result of a 31-week gestation, weighing 3.5 lbs at birth. The pregnancy was complicated by preeclampsia and administration of medication for hypertension. He remained in the NICU for 6 weeks. He experienced bradycardia and acid reflux. He had previously passed newborn hearing screenings utilizing measurements of otoacoustic emissions for both ears. An audiogram (Figure 1) was obtained which showed a unilateral hearing loss of severe degree and sensorineural origin for the left ear.

**Figure 2:**
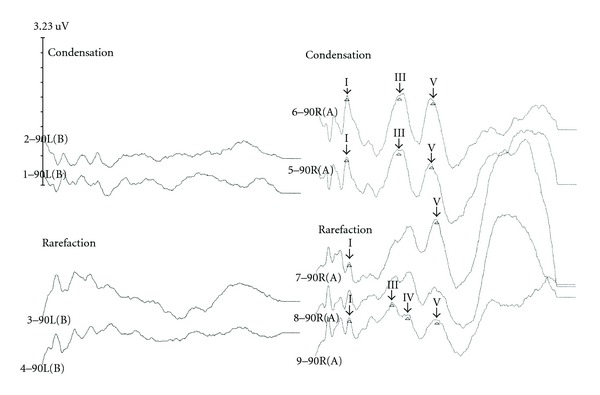
Markedly abnormal auditory brainstem response waveform morphology showing the absence of neural components with only the cochlear microphonic present in an ear with auditory neuropathy/dyssynchrony syndrome for the left ear.

CF was unable to repeat any words spoken to his left ear even at markedly elevated intensity levels. Tympanometry was within normal limits for both ears. Acoustic reflexes were present for ipsilateral stimulation of the right ear for pure tone stimuli (500–4000 Hz) but were absent at equipment limits for the left ear. Transient-evoked otoacoustic emissions were present for stimulation of both ears. Due to the discrepancy between pure tone sensitivity findings indicating the presence of a severe hearing loss, poorer speech processing than would be expected, and the presence of otoacoustic emissions, measurements of the auditory brainstem response were undertaken. The ABR showed markedly abnormal waveform morphology for stimulation of the left ear as shown in Figure 2. Neural components could not be identified at equipment limits. However, a cochlear microphonic was identified by presenting rarefaction and condensation clicks and observing a change in the phase of the early activity in the first 1–4 msec following the stimulus presentation which is depicted in Figure 2. CF was diagnosed with unilateral auditory neuropathy/dyssynchrony syndrome (ANDS) [2]. Preferential seating to the front left and use of an FM system were recommended in the educational setting.

**Case 2**

**Figure 3:**
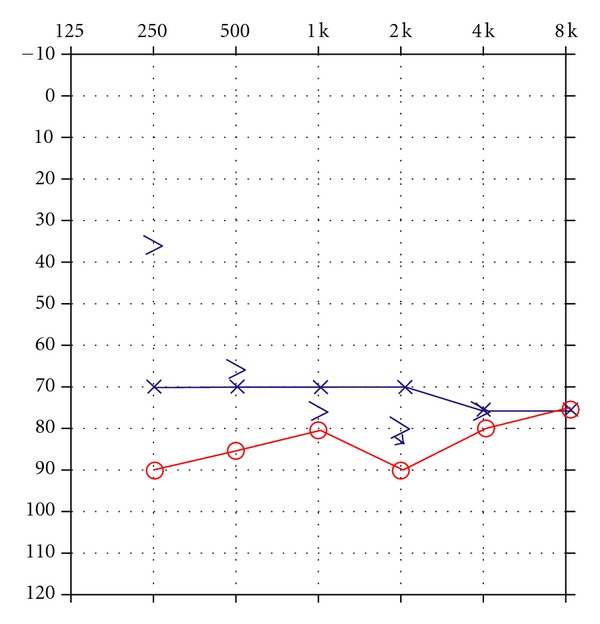
Audiogram depicting the presence of a severe hearing loss for pure tones for both ears.

GW sustained a closed head injury when he was involved in a motor vehicle accident as a restrained driver at age 23. Upon arrival at the emergency room, he was unresponsive and intubated and had a Glasgow Coma Scale of 3. An initial brain scan revealed acute subarachnoid hemorrhage involving the frontal lobes bilaterally as well as further hemorrhage into the quadrigeminal plate cistern with bilateral damage to the inferior colliculi. There was also some extension of the hemorrhage into the left lateral ventricle. In addition, GW sustained an orbital fracture. A swallow study revealed markedly abnormal oral and pharyngeal phases of swallowing with aspiration with thick puree and thin liquids. Speech was severely dysarthric and GW is confined to a wheelchair as he is unable to ambulate. An audiological evaluation revealed the presence of a severe hearing loss for pure tones for both ears as shown in Figure 3.

**Figure 4:**
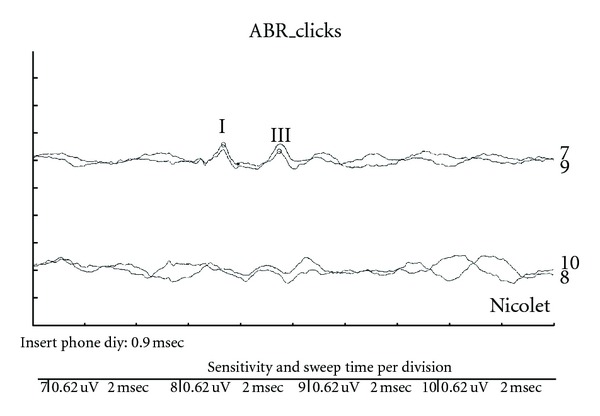
Abnormal auditory brainstem response waveform morphology consisting of an absent wave V ears in a patient with brainstem pathology secondary to closed head injury. Data are shown for ipsilateral and contralateral stimulation of the left ear.

Speech audiometry was attempted but could not be completed as the patient was unable to repeat spondaic words or point to pictures of spondaic words for stimulation of either ear. He reported that he could detect the words but could not understand any of the words. Results of ABR testing indicated the presence of abnormal waveform morphology for stimulation of both ears. ABR data are shown in Figure 4 for the left ear. Wave V was absent for all conditions. The ABR interpeak latency intervals (i.e., I–III) were within normal limits for stimulation of both ears.

**Figure 5:**
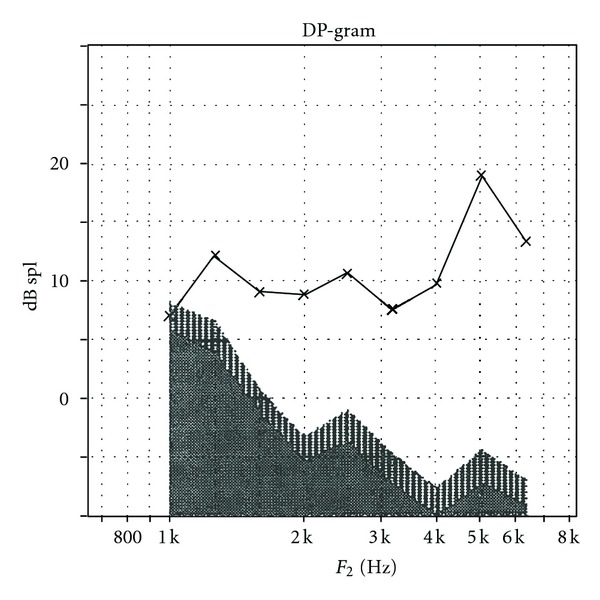
Distortion product otoacoustic emissions in a patient with brainstem pathology secondary to closed head injury for stimulation of the left ear.

Tympanometry revealed normal mobility/pressure for both ears, indicating the presence of normal middle ear function bilaterally. Acoustic reflexes were present at normal intensity levels for all stimulus conditions for ipsilateral and contralateral stimulation of both ears. Findings indicated the presence of intact structure and/or function in afferent and efferent neural elements mediating the acoustic reflex arc at brainstem levels. Measurement of transient-evoked and distortion product otoacoustic emissions were undertaken for stimulation of both ears. Robust TEOAEs and DPOAEs were present for stimulation of both ears and GW's DPOAEs are shown in Figure 5.

GW was diagnosed with a neural hearing loss secondary to bilateral damage to the inferior colliculi. Amplification was attempted but GW received no benefit from hearing aids. GW was referred to an assistive technology service for assistance in establishing a communication system.

**Case 3**

**Figure 6:**
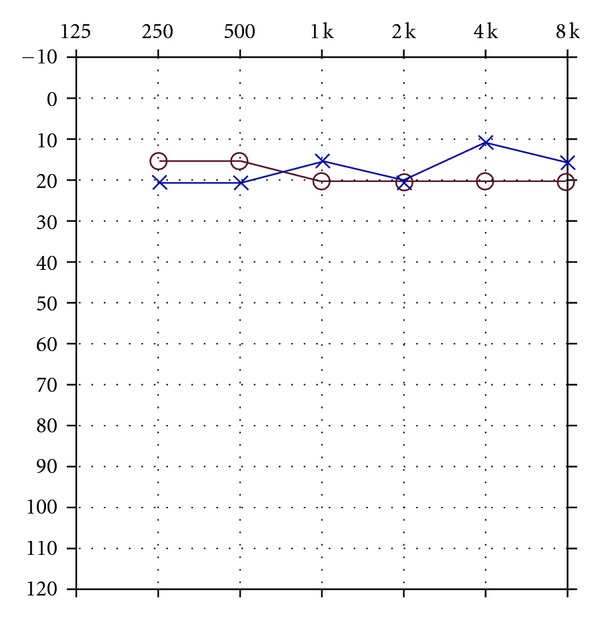
Audiogram depicting normal peripheral auditory sensitivity for both ears.

Patient CF was a normally functioning middle school age child until he was struck by a motor vehicle while riding on a bicycle. He was unresponsive at the scene of the accident with a Glasgow Coma Scale of 3. Magnetic resonance imaging undertaken 10 days after CF's injury showed extensive damage to subcortical and cortical structures including white matter edema of the subcortical medial bifrontal regions, cortical injury to the anterior temporal poles, edema to the left splenium of the corpus callosum, edema of the bilateral caudate nucleus, edema of the right thalamus, edema of the bilateral caudate nucleus, edema of the right thalamus, edema of the posterior limb of the right internal capsule, edema of the left posterior thalamus extending in the lateral midbrain, and edema of the left cerebral peduncle. Unlike the patients discussed above, CF was found to have normal peripheral auditory sensitivity (Figure 6).

**Figure 7:**
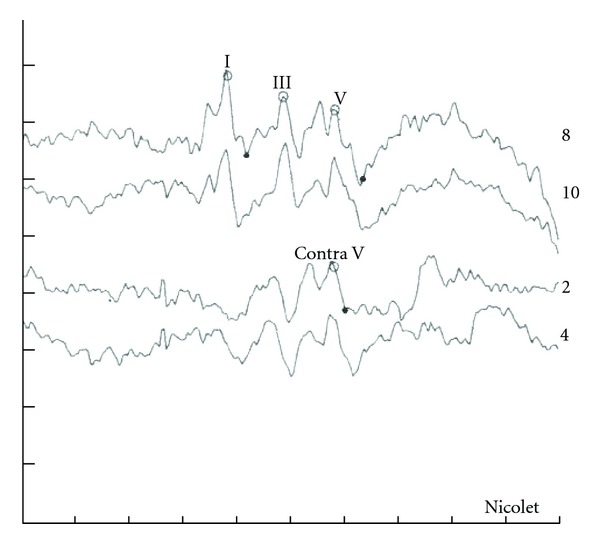
Normal auditory brainstem response morphology in a patient with auditory agnosia.

He was also found to have normal acoustic reflexes for both ipsilateral and contralateral stimulation of both ears and a normal auditory brainstem response (Figure 7).

However, the middle latency response was absent for stimulation of both ears. CF experienced marked difficulty in processing speech. He was assessed with a variety of standardized and unstandardized tests of central auditory function as well as electrophysiological measures which have been reported in more detail previously [3]. The middle latency response (MLR) was absent for stimulation of both ears. CF was diagnosed with auditory agnosia.

## 2. Discussion

All of the patients reported above had markedly impaired speech perception despite the presence of normal otoacoustic emissions which is indicative of normally functioning outer hair cells [4, 5]. For patients with sensorineural and other forms of nonconductive hearing loss, OAEs have provided a means of separating purely sensory from neural forms of hearing loss. The presence of OAEs has also provided a means to confirm the presence of retrocochlear pathology when the ABR is abnormal in pediatric neurological disorders [6] although the use of conventional audiometric procedures is a necessary adjunct to determine the functional hearing abilities of patients [7]. While measures of otoacoustic emissions did not accurately predict the extent of the communication difficulties of any of the patients, the presence of TEOAEs in conjunction with an abnormal audiogram was pivotal in pointing the need for further electrophysiologic measures to better define the auditory pathology manifested by the patients. In no case did the audiogram predict the degree of impairment in speech processing. This is most clearly evident for Case 3 in which the audiogram indicated the presence of normal peripheral auditory sensitivity for pure tones. While Cases 1 and 2 did have significant peripheral hearing loss, the impairment in speech processing was greater than expected given the pure tone findings [8]. ABR measurements did suggest the presence of ANDS for Case 1 and ABR findings were consistent with the lesions to the inferior colliculi for Case 2 but were insensitive to the subcortical and cortical level deficits manifested in Case 3.

The auditory brainstem response was instrumental in diagnosis of the mechanisms underlying the hearing and speech processing deficits for Cases 1 and 2. The ABR relies on the synchronous discharge of neural units in the auditory pathways from the 8th nerve through the auditory pathway in the brainstem. On the basis of measurements obtained during operations for cranial nerve disorders in humans [9], it has been determined that the neural generator for waves I and II in humans is the auditory nerve. It is more difficult to attribute specific generators to the later peaks of the ABR due to the extent of parallel processing in the auditory pathway at brainstem levels; the peaks of waves after wave II have multiple sources underlying their generation [10]. While there is evidence on the basis of intraoperative recordings that the neural generator for wave III of the human ABR is the cochlear nucleus [11] and that the generation of wave III may be more complex than originally supposed. The contralateral cochlear nucleus [12] as well as the most proximal portion of the auditory nerve may also make a contribution to the generation of wave III [13]. Several neural sources contribute as the generators for wave V [14]. The most positive peak of wave V is probably generated at the termination of the fiber tract of the lateral lemniscus, whereas the following negative trough in conventionally recorded ABRs is generated by slow dendritic potentials in the inferior colliculus [14]. The main contribution to the peak of wave V appears to be from contralateral rather than ipsilateral structures on the basis of intracranial recordings in human subjects [12]. Neural components in the ABR were absent for Case 1 either because of failure of the cochlea to adequately stimulate the auditory nerve or a deficit in the auditory nerve itself. For Case 2, the absence of wave V for stimulation of both ears was consistent with the presence of bilateral lesions to the inferior colliculi. The absence of ABR abnormalities for Case 3 suggested that the auditory pathways through brainstem levels were intact.

Measurements of the acoustic reflex were also insensitive to the auditory pathology manifested for Cases 2 and 3 but were consistent with the presence of the unilateral deficit present for Case 1. The acoustic reflex in humans involves the contraction of the stapedius muscle in response to sounds of moderate to high intensity. The reflex arc involves the neuronal connections between the cochlear nucleus on one side of the brainstem and the motor nuclei of cranial nerve VII bilaterally and the stapedius muscle on each side [15]. The afferent component begins with the cochlear branch of the 8th nerve which provides input to the ventral cochlear nucleus (VNC). There are neural pathways from the VCN to each superior olivary complex (SOC). From the ipsilateral and contralateral SOC, neural input is directed to the motor nuclei of the 7th nerve which innervates the stapedius muscle on each side. The efferent portion of the acoustic reflex involves the VIIth cranial nerves and the stapedius muscle. Lesions in afferent or efferent components of the acoustic reflex arc can result in abnormal thresholds. In the affected ear in Case 1, the acoustic reflexes were absent given that ANSD is associated with failure of the cochlea to excite the 8th nerve to respond or to dysfunction to the 8th nerve itself. 

The diagnosis of ANSD for the left ear in Case 1 stems from the presence of hearing loss, disproportionate loss of speech discrimination skills, and absence of acoustic reflexes with present TEOAEs. Risk factors for ANSD include prematurity, hyperbilirubinemia, and history of administration of ototoxic medications although 38.5% of cases report normal birth and neonatal history [2]. Speech processing ability in ANSD is typically poor [16]. The audiogram for pure tones can range from normal to profound hearing loss. ANSD is present in up to 40% of NICU graduates with hearing loss [17]. A number of studies have previously reported on the presence of unilateral ANSD [18–20]. As noted by Berlin et al. [2], the majority of cases of unilateral ANSD have involved the left ear (13/19) which is also the case for Case 1. The mechanisms underlying ANSD in an alive patient cannot be established with certainty as the cochlea and auditory nerve are not available for histological evaluation. The dysfunction in ANSD may reflect impairment involving the inner hair cells of the cochlea, possible impairment in the synapse between the inner hair cells and the auditory nerve, impairment in the ganglion neurons, or impairment in the auditory nerve itself [16]. The presence of TEOAEs indicates only that the outer hair cells in the cochlea are intact. When the site of the impairment is in the auditory nerve itself, the presence of cochlear nerve deficiency can be documented with inclined sagittal MRI of the internal canal in unilateral ANSD [21].

Hearing loss resulting from lesions to the inferior colliculi secondary to head trauma has been reported previously [22–24]. The majority of cases with hearing loss and damage to the inferior colliculi have reflected bilateral involvement [22–26] although unilateral involvement has also been reported [27]. Lesions to the inferior colliculi would be expected to have a devastating impact on auditory function for, as noted by Musiek and Baran [28], most of the auditory fibers from the lateral lemniscus and lower nuclei synaspe either directly or indirectly with the inferior colliculus.

The presence of acoustic reflexes for Case 2 is consistent with the presence of ABR components I and III and the midbrain level of the pathology. The intact contralateral acoustic reflexes suggest that the auditory pathway through the level of the pons, including the trapezoid body, is intact. Intact acoustic reflexes have been reported previously in patients with lesions to the inferior colliculi [23, 24, 29] although an exception to this trend has been reported [22]. In a patient with bilateral midbrain contusion, preserved ipsilateral acoustic reflexes with absent reflexes for contralateral stimulation have been reported [22]. This was attributable to damage to the trapezoid body with sparing of the superior olivary complex [22] (see comments by A. Moller). Several studies have reported the presence of normal ABRs in patients with lesions to the inferior colliculi, [23–25] while other studies have reported bilateral absence or significant reduction in amplitude of wave V in patients with midbrain damage to both inferior colliculi [22, 29]. Normal sensitivity for pure tones or only mild-moderate hearing loss has been reported in several patients with inferior collicular lesions [25, 26], whereas for Case 2 and other patients the pure tone audiogram has shown a severe hearing loss or was unobtainable due to patients', inability to respond consistently to stimuli [22, 23]. All of the studies agree with respect to the presence of severe speech processing deficits in patients with lesions to the inferior colliculi although for some patients their impairments have been temporary and resolved after 3 or 18 months [24]. Case 2's speech processing has not improved over an 8-year period following his injury.

The mechanism underlying the speech processing deficits for Case 3 was the presence of subcortical and cortical level damage sustained to auditory structure and function following a traumatic brain injury [3]. Peripheral aspects of auditory function such as peripheral auditory sensitivity, TEOAEs, acoustic reflexes, and the ABR were entirely preserved, while speech processing was markedly impaired.

All of the patients reported upon in the present paper experienced severe speech processing deficits which were bilateral for Cases 2 and 3 and unilateral for Case 1. Measurements of the MLR were instrumental in defining the source of the deficits experienced by Case 3 who was diagnosed with auditory agnosia. Absent MLRs in the presence of normal peripheral auditory sensitivity is consistent with the presence of bilateral lesions of auditory radiations and/or bilateral lesions in the auditory cortex [30]. The neural generators of the MLR include not only units in the thalamocortical pathway but aspects of units in the later developing mesencephalic reticular formation [31, 32]. The presence of higher cortical dysfunction impacts on the MLR [33]. The MLR is sensitive to interaural level and timing differences which suggest that the MLR is associated with the neural mechanisms underlying processes of sound localization [34].

The cases reviewed in the present paper have a number of implications for audiological practice. Use of otoacoustic emissions for screening of newborns will not identify ANSD in newborns. Given the increased prevalence of ANSD in NICU graduates, the use of ABR screening methods in the NICU is advisable. Comprehensive audiological assessment is recommended in the presence of delays in the acquisition of speech and language skills or failed screenings despite the fact that a child has passed a newborn hearing screening. Provision of hearing aids for patients with auditory disorders as that manifested by Case 2 is inadvisable. Not only was there a lack of aided benefit for Case 2 but the patient also had loudness processing issues for amplified speech; furthermore, amplified speech was no clearer for the patient. The outcome of an amplification trial was predictable on the basis of poor performance for processing for speech 20–30 dB HL above Case 2's threshold for speech. The auditory deficits of Case 3 were initially missed by administration of a protocol that correctly identified the presence of normal peripheral auditory sensitivity for pure tones, normal acoustic reflexes, and normal OAEs. Only after several disciplines treating the patient (i.e., neuropsychology, speech pathology) questioned the adequacy of the patient's hearing was a more comprehensive battery of tests administered and his auditory agnosia identified.

## References

[1] Berlin CI, Hood LJ, Jeanfreau J, Morlet T, Brashears S, Keats B, Berlin CI, Hood LJ, Ricci A (2002). The physiological bases of audiological management. *Hair Cell Micromechanisms and Otoacoustic Emissions: Thomson-Delmar Learning*.

[2] Berlin CI, Hood LJ, Morlet T (2010). Multi-site diagnosis and management of 260 patients with auditory neuropathy/dys-synchrony (auditory neuropathy spectrum disorder). *International Journal of Audiology*.

[3] Hattiangadi N, Pillion JP, Slomine B, Christensen J, Trovato MK, Speedie LJ (2005). Characteristics of auditory agnosia in a child with severe traumatic brain injury: a case report. *Brain and Language*.

[4] Kemp DT (1978). Stimulated acoustic emissions from within the human auditory system. *Journal of the Acoustical Society of America*.

[5] Kemp DT (1986). Otoacoustic emisions, travelling waves and cochlear mechanisms. *Hearing Research*.

[6] Ferber-Viart C, Duclaux R, Dubreuil C, Sevin F, Collet L, Berthier JC (1994). Otoacoustic emissions and brainstem auditory evoked potentials in children with neurological afflictions. *Brain and Development*.

[7] Kon K, Inagaki M, Kaga M, Sasaki M, Hanaoka S (2000). Otoacoustic emission in patients with neurological disorders who have auditory brainstem response abnormality. *Brain and Development*.

[8] Yellin MW, Jerger J, Fifer RC (1989). Norms for disproportionate loss in speech intelligibility. *Ear and Hearing*.

[9] Moller AR, Jannetta PJ, Moller MB (1981). Neural generators of brainstem evoked potentials. Results from human intracranial recordings. *Annals of Otology, Rhinology and Laryngology*.

[10] Moller AR (1995). *Interoperative Neurophysiologic Monitoring*.

[11] Moller AR, Jannetta P, Moller MB (1982). Intracranially recorded auditory nerve response in man. New interpretations of BSER. *Archives of Otolaryngology*.

[12] Moller AR, Jho HD, Yokota M, Jannetta PJ (1995). Contribution from crossed and uncrossed brainstem structures to the brainstem auditory evoked potentials: a study in humans. *Laryngoscope*.

[13] Moller AR, Jho HD (1991). Compound action potentials recorded from the intracranial portion of the auditory nerve in man: effects of stimulus intensity and polarity. *Audiology*.

[14] Moller AR, Jannetta PJ (1982). Evoked potentials from the inferior colliculus in man. *Electroencephalography and Clinical Neurophysiology*.

[15] Borg E (1974). On the neuronal organization of the acoustic middle ear reflex. *Archives of Otolaryngology*.

[16] Starr A, Picton TW, Sininger Y, Hood LJ, Berlin CI (1996). Auditory neuropathy. *Brain*.

[17] Rea PA, Gibson WPR (2003). Evidence for surviving outer hair cell function in congenitally deaf ears. *Laryngoscope*.

[18] Konrádsson KS (1996). Bilaterally preserved otoacoustic emissions in four children with profound idiopathic unilateral sensorineural hearing loss. *Audiology*.

[19] Podwall A, Podwall D, Gordon TG, Lamendola P, Gold AP (2002). Unilateral auditory neuropathy: case study. *Journal of Child Neurology*.

[20] Buchman CA, Roush PA, Teagle HFB, Brown CJ, Zdanski CJ, Grose JH (2006). Auditory neuropathy characteristics in children with cochlear nerve deficiency. *Ear and Hearing*.

[21] Liu C, Bu X, Wu F, Xing G (2012). Unilateral auditory neuropathy caused by cochlear nerve deficiency. *International Journal of Otolaryngology*.

[22] Jani NN, Laureno R, Mark AS, Brewer CC (1991). Deafness after bilateral midbrain contusion: a correlation of magnetic resonance imaging with auditory brain stem evoked responses. *Neurosurgery*.

[23] Hu CJ, Chan KY, Lin TJ, Hsiao SH, Chang YM, Sung SM (1997). Traumatic brainstem deafness with normal brainstem auditory evoked potentials. *Neurology*.

[24] Vitte E, Tankéré F, Bernat I, Zouaoui A, Lamas G, Soudant J (2002). Midbrain deafness with normal brainstem auditory evoked potentials. *Neurology*.

[25] Meyer B, Kral T, Zentner J (1996). Pure word deafness after resection of a tectal plate glioma with preservation of wave V of brain stem auditory evoked potentials.. *Journal of Neurology, Neurosurgery, and Psychiatry*.

[26] Hoistad DL, Hain TC (2003). Central hearing loss with a bilateral inferior colliculus lesion. *Audiology and Neuro-Otology*.

[27] Pawar SJ, Sharma RR, Karapurkar AP, Tewari MK, Lad SD (2003). Angiolipoma of the right inferior colliculus: a rare central cause of hearing loss and limb ataxia. *Journal of Clinical Neuroscience*.

[28] Musiek FE, Baran JA (1986). Neuroanatomy, neurophysiology, and central auditory assessment. Part I: brain stem. *Ear and Hearing*.

[29] Musiek FE, Charette L, Morse D, Baran JA (2004). Central deafness associated with a midbrain lesion. *Journal of the American Academy of Audiology*.

[30] Scherg M, Von Cramon D (1986). Evoked dipole source potentials of the human auditory cortex. *Electroencephalography and Clinical Neurophysiology*.

[31] Kraus N, McGee T (1990). Clinical applications of the middle latency response. *Journal of the American Academy of Audiology*.

[32] Kraus N, McGee T, Littman T, Nicol T (1992). Reticular formation influences on primary and non-primary auditory pathways as reflected by the middle latency response. *Brain Research*.

[33] Kraus N, Ozdamar O, Hier D, Stein L (1982). Auditory middle latency responses (MLRs) in patients with cortical lesions. *Electroencephalography and Clinical Neurophysiology*.

[34] Leigh-Paffenroth ED, Roup CM, Noe CM (2011). Behavioral and electrophysiologic binaural processing in persons with symmetric hearing loss. *Journal of the American Academy of Audiology*.

